# Sensing of Dietary Lipids by Enterocytes: A New Role for SR-BI/CLA-1

**DOI:** 10.1371/journal.pone.0004278

**Published:** 2009-01-26

**Authors:** Olivier Béaslas, Carine Cueille, François Delers, Danielle Chateau, Jean Chambaz, Monique Rousset, Véronique Carrière

**Affiliations:** 1 Université Pierre et Marie Curie - Paris 6, UMR S 872, Les Cordeliers, Paris, France; 2 INSERM, U 872, Paris, France; 3 Université Paris Descartes, UMR S 872, Paris, France; The Research Institute for Children at Children's Hospital New Orleans, United States of America

## Abstract

**Background:**

The intestine is responsible for absorbing dietary lipids and delivering them to the organism as triglyceride-rich lipoproteins (TRL). It is important to determine how this process is regulated in enterocytes, the absorptive cells of the intestine, as prolonged postprandial hypertriglyceridemia is a known risk factor for atherosclerosis. During the postprandial period, dietary lipids, mostly triglycerides (TG) hydrolyzed by pancreatic enzymes, are combined with bile products and reach the apical membrane of enterocytes as postprandial micelles (PPM). Our aim was to determine whether these micelles induce, in enterocytes, specific early cell signaling events that could control the processes leading to TRL secretion.

**Methodology/Principal Findings:**

The effects of supplying PPM to the apex of Caco-2/TC7 enterocytes were analyzed. Micelles devoid of TG hydrolysis products, like those present in the intestinal lumen in the interprandial period, were used as controls. The apical delivery of PPM specifically induced a number of cellular events that are not induced by interprandial micelles. These early events included the trafficking of apolipoprotein B, a structural component of TRL, from apical towards secretory domains, and the rapid, dose-dependent activation of ERK and p38MAPK. PPM supply induced the scavenger receptor SR-BI/CLA-1 to cluster at the apical brush border membrane and to move from non-raft to raft domains. Competition, inhibition or knockdown of SR-BI/CLA-1 impaired the PPM-dependent apoB trafficking and ERK activation.

**Conclusions/Significance:**

These results are the first evidence that enterocytes specifically sense postprandial dietary lipid-containing micelles. SR-BI/CLA-1 is involved in this process and could be a target for further study with a view to modifying intestinal TRL secretion early in the control pathway.

## Introduction

The increased incidence of metabolic disorders (obesity, metabolic syndromes and diabetes) and the ensuing atherosclerosis and cardiovascular diseases are linked to the significant changes in dietary habits that have occurred in recent decades, among which is an increase in fat intake [1]. Marked and prolonged postprandial hypertriglyceridemia, characterized by the accumulation of apolipoprotein B-containing triglyceride-rich lipoproteins (TRL), is a significant contributor to the development of dyslipidemia and a known risk factor for atherosclerosis [2]. Enterocytes in the intestine, the first organ to come into contact with digestion products, transfer dietary lipids to the organism and largely contribute to the production of TRL. It is thus important to characterize the mechanisms involved in the control of lipid absorption in these cells, especially those modulating the synthesis and secretion of TRL, as this could lead to the development of drugs acting on the early control steps in the intestinal transfer of dietary lipids, which could be used to reduce postprandial hypertriglyceridemia.

The absorption of lipids through the intestine is no longer considered a passive process but rather an active regulation of highly polarized mechanisms [3]. During the postprandial period, dietary lipids, mostly triglycerides (TG), after being hydrolyzed by pancreatic enzymes into fatty acids (FA) and monoglycerides (MG) and solubilized by bile salts and lipids in the intestinal lumen, are supplied to enterocytes as postprandial micelles (PPM). After absorption of FA and MG by enterocytes, TG must be re-synthesized in the endoplasmic reticulum and associate with the structural apolipoprotein (apo) B48, and apoA-I and apoA-IV to produce chylomicrons, the intestinal postprandial form of TRL that are secreted into lymph and then into the general circulation [3], [4]. The small intestine must adapt to the variations in lipid load and composition that occur daily between postprandial and interprandial periods (for review [5]). The adaptation of enterocyte function has mainly been studied in terms of the effect of dietary lipids on gene expression and the action of transcription factors (for review [6]).

At the same time, the small intestine signals nutrient abundance to the organism and contributes to satiety by the release of hormones and enteropeptides from enteroendocrine cells and by the secretion of chylomicrons and apoA-IV, a satiety signal [7], by enterocytes (for reviews [8], [9]). However, most studies on the effects of dietary lipids in the small intestine do not relate the structural aspects of lipid supply to enterocytes to the specificity of the effects. More precisely, they do not address whether the ‘physiological’ mode of delivering dietary lipids to the apical pole of enterocytes as complex micelles, known to be critical for intestinal lipid intake [10], is required to induce the effects reported. While some mechanisms by which enteroendocrine cells sense lipids have been described [11], it is not known whether enterocytes sense dietary lipids. Previous works from our group indicated that there is some sensing of micellar dietary lipids by enterocytes as they demonstrated that the apical supply of postprandial micelles (mimicking those present in the intestinal lumen *in vivo* after a meal) induced specific effects in Caco-2/TC7 cells, a model reproducing in culture most of the morphological and functional characteristics of enterocytes [12], [13]. These effects involved the rapid displacement of the apical brush-border-associated pool of apoB towards intracellular secretory compartments [14] and the intracellular neosynthesis of TG, leading to the secretion of TRL [15]. In addition, the apical supply of PPM induced the activation of apoA-IV transcription via the transcription factor HNF-4 [16], a process also recently reported to occur in pig enterocytes *in vivo* [17]. These PPM-induced effects were not obtained by the apical supply of albumin-bound fatty acids or of PPM lacking some component(s) or by supplying plasma fatty acids at the base of cells [14]–[16]. These results indicated that the apical supply of fatty acids was not sufficient to trigger the cascade of events leading to the secretion of TRL and suggested that TRL secretion could involve an initial step whereby complex dietary lipid-containing micelles are sensed via a receptor located at the apical membrane of enterocytes, which would induce specific signals.

Our aim was thus to analyze the early events that are specifically induced by the apical supply of PPM and to determine the molecular mechanisms of PPM sensing in enterocytes.

## Materials and Methods

### Chemicals and antibodies

Unless indicated, all chemicals were purchased from Sigma France. Antibodies used were: mouse polyclonal anti-Cla-1 (BD Transduction Laboratories), rabbit polyclonal anti-SR-BI (Novus 400-104), sheep polyclonal anti-PLAP (ABDSerotec 0300-1909), rabbit polyclonal anti-ERK (Cell Signaling), rabbit polyclonal anti-P-ERK (Thr202/Tyr204, Cell Signaling), rabbit polyclonal anti-p38 MAPK (Cell Signaling), rabbit polyclonal anti-P-p38 MAPK (Thr180/Tyr182, Cell Signaling), mouse monoclonal anti-E-cadh (BD transduction laboratories), goat polyclonal anti-EEA1 (Santa Cruz Biotechnology), mouse monoclonal anti-flotillin-1 (BD Transduction Laboratories), mouse monoclonal anti-sucrase-isomaltase (provided by Dr. Swallow, University College London) and goat polyclonal anti-apoB (Immuno France). Secondary Cy3 and Cy2 dye-labeled antibodies were from Jackson ImmunoResearch Laboratories. Peroxidase-conjugated immunoglobulins were from Biosys France. Alexa 488-conjugated Concanavalin A was from Molecular Probes.

### Cell culture and stable knockdown of SR-BI/CLA-1 gene expression by shRNA

Caco-2/TC7 cells [12] were cultured on semi-permeable filters as described [14]. All experiments were performed 21 days after seeding, i.e. when the cells have acquired their optimal capacity of TRL secretion [15]. In some experiments, cycloheximide was added 1 hour before the treatment of the cells and during all the time of experiment, as already reported [14]. For stable SR-BI/CLA-1 knockdown experiments, lentiviral particles containing short hairpin RNA (shRNA) sequences targeted to human SR-BI/CLA-1 (GenBank accession number NM_005505) were purchased from Sigma MISSION™ Lentiviral Transduction Particles. Two lentiviral vectors containing shRNA sequences identified in The RNAi Consortium shRNA library as TRCN0000056963 (shRNA 63) and TRCN0000056964 (shRNA 64) were used and their transfection in Caco-2/TC7 cells gave Cell populations 63 and 64, respectively. One day after seeding on 24-well culture plates (Corning, France), dividing Caco-2/TC7 cells were incubated overnight with 8 µg/ml hexadimethrine bromide and 200 µl of shRNA lentiviral particles containing 10^6^ lentiviral transducing particles/ml in DMEM-FBS. The medium was then changed daily. The SR-BI/CLA-1 knockdown cells were able to grow and to differentiate as well as untransfected Caco-2/TC7 cells (data not shown). Stably transfected cells were selected with 10 µg/ml puromycin. Total puromycin-resistant cell populations were analyzed. Stock SR-BI/CLA-1 knockdown cell populations were cultured in the presence of puromycin to sustain the selection. Whatever the culture passages that were analyzed, all cell populations remained resistant to puromycin. For maintenance of infected cell populations, a 6-day passage frequency was used. The experiments were performed after 21 days of culture on semi-permeable filters, without puromycin.

### Preparation of lipid micelles-containing media

Preparation of lipid micelles was performed as described [15] The composition of postprandial lipid micelles (PPM) was similar to that of human duodenum lumen [18] after a lipid-rich meal (0.6 mM oleic acid, 0.2 mM L-α-lysophosphatidylcholine, 0.05 mM cholesterol, 0.2 mM 2-monooleoylglycerol, 2 mM taurocholic acid). Inter-prandial lipid micelles (IPM) were prepared as PPM but, according to physiological conditions [19], were devoid of oleic acid and 2-monooleoylglycerol. PPM or IPM were added to the medium of the upper compartment for the last day of culture, for the indicated times.

### Competitors and inhibitors of SR-BI/CLA-1 and of protein kinases

HDL_3_ were isolated by ultracentrifugation as described [20], from EDTA-plasma obtained from human volunteers, supplemented with 0.005% gentamycin, 0.04% sodium azide and protease inhibitors. HDL_3_ (100 µg/ml) were added in the apical medium at the same time as PPM.

For studies with inhibitors, Caco-2/TC7 cells were pre-incubated 1 hour in the presence of 20 µM of the selective inhibitor of P38 MAPK, SB203580 (Calbiochem), or 10 µM of BLT-1 (Chembridge corporation, USA), an inhibitor of SR-BI-dependent lipid transport, in both apical and basal compartments. Fresh solutions of inhibitors were then added in the apical medium at the same time as PPM. Both inhibitors were diluted in DMSO.

### Confocal Fluorescence Microscopy

Immunoflorescence and confocal analyses were performed as described [14]. Cells were fixed with 4% paraformaldehyde (wt/vol) and permeabilized with 0.2% Triton X-100 for apoB or 0.01% saponin for SR-BI/CLA-1, at 4°C. In some experiments, alexa 488-conjugated concanavalin A was added at the same time as primary antibodies to visualize the apical brush-border. Immunofluorescence was examined by confocal fluorescence microscopy (LSM510 microscope Zeiss Germany, with a 63x water lens, 80 µm pinhole). Images were treated using Photoshop and Illustrator software (Adobe).

### Cell surface biotinylation assay

Caco-2/TC7 cells cultured on filters were incubated without or with PPM, and washed with ice-cold PBS containing 1 mM CaCl_2_ and 0.5 mM MgCl_2_. Cells were then incubated (30 min, 4°C) with 0.9 mg/ml sulfo-NHS-SS-Biotin, added only to the apical compartment. Apical membrane expression of SR-BI/CLA-1 was analyzed by biotin labeling of surface proteins with EZ-Link Sulfo-NHS-SS-Biotin, followed by immunoprecipitation with immobilized streptavidin using the Cell Surface Protein Biotinylation and Purification Kit (Pierce), according to manufacturer's instructions. Total cell lysates obtained before immunoprecipitation (total), immunoprecipitated fractions containing apical biotinylated proteins (apical fraction) and non-immunoprecipitated fractions containing unlabeled proteins (non apical fraction), were subjected to immunoblot analyses. In some experiments sulfo-NHS-SS-Biotin was added only in the basal compartment and the basal fraction containing biotinylated proteins was recovered as described above.

### Sucrose gradient

For sucrose density gradients, 10^8^ Caco-2/TC7 cells were homogenized on ice in 2 ml of 10 mM Tris-HCl, pH 8, 150 mM NaCl buffer (TBS) containing 1% Triton X-100 and protease and phosphatase inhibitor cocktails. Homogenate was adjusted to 40% sucrose by 2 ml of 80% sucrose/TBS. The resulting 4 ml were covered with 4 ml of 30% sucrose and 4 ml of 5% sucrose and centrifuged (SW41, L8 Beckman, 18h, 39,000 rpm, 4°C). Sequential 1 ml fractions were collected from the top of the tube. Eleven fractions were obtained, to which NP-40 was added (1% final concentration).

### Ligand blotting assay with micelles

Homogenates from differentiated Caco-2/TC7 cells were prepared in Tris/mannitol buffer (2 mM Tris-HCl pH 7.4, 50 mM mannitol, 2% protease inhibitor coktail) and centrifuged for 15 min (5,000 rpm, 4°C). Supernatants were subjected to SDS-PAGE. Proteins were transfered onto Bio-Rad nitrocellulose membranes, which were saturated for 30 min with 6% defated milk in PBS containing 0.25 M NaCl (PBS/NaCl). For lipid overlay, 0.05 mM biotinylated phosphatidylethanolamine was incorporated to the preparation of PPM or IPM with a 1/200 molar ratio toward L-α-lysophosphatidylcholine. Micelles were mixed with coupled horseradish-streptavidin peroxydase (1 µg/ml, Jackson ImmunoResearch) and incubated 10 min at room temperature. Membranes containing blotted proteins were incubated with this solution for 2 hours at room temperature, washed with PBS/NaCl and revealed by ECL system.

For immunoprecipitation of SR-BI/CLA-1, 5 µl of rabbit anti-SR-BI antibodies (Novus NB400-104) or rabbit non-immune serum were mixed with 25 µl of 400 mg/ml protein A-sepharose (Healthcare Biosciences) in Tris buffer (20 mM Tris-HCl, pH 7.4, 5 mM EDTA, 0.15 M NaCl, 5 mM lactose and 0.1% ovalbumin) for 1 hour at room temperature. After several washings with Tris buffer, SR-BI antibodies combined to protein A-sepharose were incubated with 90 µg of Caco-2/TC7 cell homogenates, for 2 hours at 4°C. After several washings with Tris buffer, immunoprecipitated material was recovered with Laemmli buffer, subjected to SDS-PAGE and then to lipid overlay.

### Western blot analysis

Caco-2/TC7 cells were scraped into lysis buffer (20 mM Tris-HCl, pH 7.4, 5 mM EDTA, 0.15 M NaCl, 1% Triton X-100, 0.5% sodium deoxycholate) containing protease and phosphatase inhibitor cocktails. Aliquots of cell lysates (40–80 µg) were subjected to SDS-PAGE under reducing conditions in a 7.5% polyacrylamide gel. Proteins were transferred onto nitrocellulose membranes, developed using ECL Western blotting reagents according to the manufacturer's instructions (Amersham Biosciences, Orsay, France) and quantified using Image J software. The levels of phospho-ERK and phospho-p38MAPK were normalized with total ERK and p38MAPK, respectively.

### Phosphoprotein screens

Caco-2/TC7 cell lysates were analyzed by Kinetworks™ KPSS-9.0 phosphorylation screens (Kinexus Bioinformatics Corp., Canada). Kinetworks analysis, performed by Kinexus, involved resolution of a single lysate sample by SDS-PAGE with 20-lane multi-immunoblotters and subsequent immunoblotting using specific antibodies against 37 phosphoproteins. Kinexus performed all the normalization and statistical analyses on the data.

### Immunoelectron microscopy

Differentiated Caco-2/TC7 cells were fixed with 4% PFA plus 0.1% glutaraldehyde. After alcohol-graded dehydration, sections were embedded in LR White, and ultra-thin sections were incubated with rabbit anti SR-BI/CLA-1 antibody (Novus, NB400-104), gold (12 nm particles)-labeled donkey anti-rabbit IgGs (Jackson Immunoresearch) being used as secondary antibodies. Double labeling of SR-BI/CLA-1 and alkaline phosphatase (PLAP) was performed by using secondary antibodies labeled with 18 nm and 12 nm gold particles, respectively. Sections were analyzed in a Jeol 100 CX II electron microscope.

### Statistical analyses

Statistical analyses were performed using Student's *t* test for unpaired data.

## Results

### Cell signaling events are specifically induced in enterocytes by postprandial micelles

**Figure 1:**
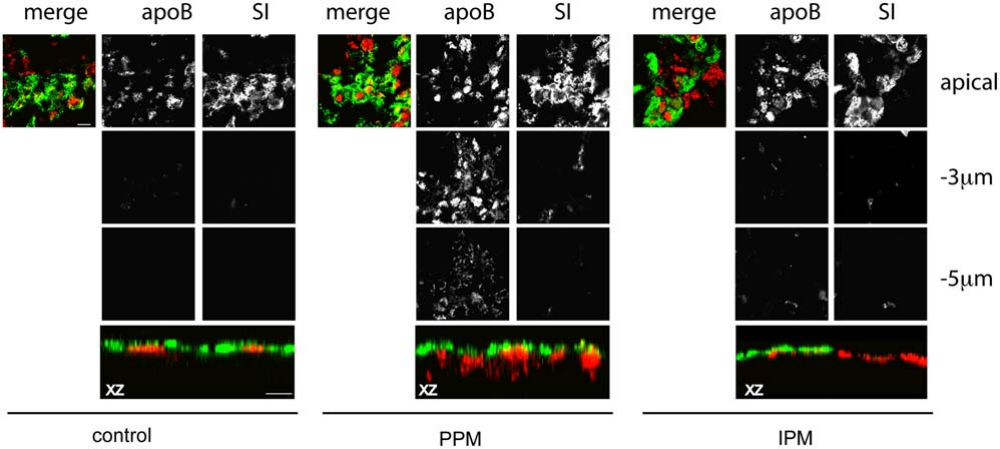
Trafficking of apoB is specifically induced by alimentary lipid-containing micelles. Confocal image of apolipoprotein B (apoB, red channel) and sucrase-isomaltase (SI, used as a brush-border marker, green channel) localizations in differentiated Caco-2/TC7 cells cultured in the absence (control) or presence of postprandial (PPM) or inter-prandial (IPM) micelles added at the apical pole of the cells for 20 min. Top panels show XY acquisitions at the apical level and at 3 µm and 5 µm below the apical plane. Bottom panels show the corresponding XZ projections (zoom ×2). (bar, 20 µm).

**Figure 2:**
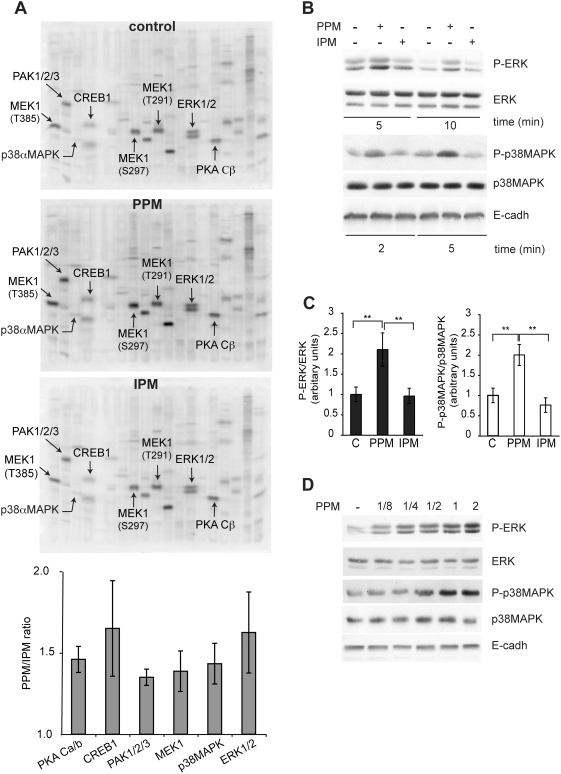
Signaling pathways specifically induced by postprandial micelles. (A) Kinetworks™ phosphoprotein immunoblots from lysates of Caco-2/TC7 cells cultured on filters without micelles (control) or with PPM or IPM for 5 min. Duplicate immunoblots using specific antibodies directed against phosphoproteins were analyzed by Kinexus. Bands corresponding to phosphoproteins specifically up-regulated by PPM are indicated by arrows. The antibodies recognized the phosphosites: S144/S141/S154 for PAK1/2/3, T180/Y182 for p38αMAPK, S129/S133 for CREB1, T202/Y204 for ERK1, T185/Y187 for ERK2 and S338 for PKA Cβ. The phosphosites analyzed for MEK1 are indicated in the figure. Histograms show the amounts of phosphoproteins expressed as the ratio of phosphorylation in the PPM versus the IPM sample (from three independent experiments). (B) Caco-2/TC7 cells cultured on semi-permeable filters were incubated in the absence or presence of PPM or IPM in the apical compartment for the indicated times. Cell lysates were analyzed by immunoblot with antibodies against phospho-ERK1/2 (P-ERK), and phospho-p38MAPK (P-p38 MAPK). Total ERK, p38MAPK and E-cadherin (E-cadh) were used as loading controls. (C) Quantification of normalized P-ERK and P-p38MAPK levels (from three independent experiments) in the absence (c) or presence of PPM or IPM for 10 min (for P-ERK and ERK) or for 5 min (for P-p38MAPK and p38MAPK), **p<0.01. (D) Caco-2/TC7 cells were cultured in the presence of various amounts of PPM supplied in the apical compartment for 10 min. Cell lysates were analyzed by immunoblot with the same antibodies as in (B) and a blot representative of three independent analyses is shown.

We previously showed that addition of PPM to Caco-2 enterocytes caused the chase of apoB from the apical brush-border towards basolateral secretory domains within 15 min [14]. To evaluate the specific effect of alimentary lipids supplied as ‘physiological’ complex PPM, we compared this effect on apoB trafficking to that induced by interprandial micelles (IPM) devoid of digested TG products (**Figure 1**). In the absence of micelles from the apical compartment, apoB co-localized with the brush-border marker sucrase-isomaltase (SI). After PPM were delivered to the apical pole, apoB was chased towards intracellular compartments, as previously reported [14]. This delocalization, which was not observed after IPM supply (**Figure 1**), indicates that the mobilization of apical apoB depends specifically on the supply of micellar alimentary lipids leading us to hypothesize that PPM can induce specific signaling pathways in enterocytes. We therefore tested whether any of multiple signaling proteins, mainly kinases, were activated using a specific, semi-quantitative method to identify them with Kinetworks™ screens. Time-course experiments were performed 5, 10 and 15 min after the apical supply of PPM or IPM. As early as 5 min after PPM supply, we observed an increase in the phosphorylation of five protein kinases, namely PAK1/2/3, MEK1, p38α MAPK, ERK1/2 and PKA Cβ (**Figure 2A**). Of these, MEK1, responsible for the activation of ERK1/2 in the canonical ERK pathway, was phosphorylated at three phosphosites (T385, S297 and T291). There was also an increase in the phosphorylation of the CREB1 protein, a target transcription factor of MAPK pathway (for review [21]). None of these effects were observed when IPM was supplied to cells (**Figure 2A**) even after 15 min (data not shown). As most of the kinases that were activated after PPM supply belong to the MAPK signaling pathway, we focused on ERK1/2 and p38 MAPK, two kinases downstream in this pathway. PPM supply specifically increased the phosphorylation of ERK1/2 and p38 MAPK by 5 min and 2 min respectively (**Figure 2B and 2C**) in a dose-dependent manner (**Figure 2D**) as determined by immunoblotting.

### The supply of PPM specifically regulates the subcellular distribution of SR-BI/CLA-1

We hypothesized that a protein located at the apical membrane of enterocytes could trigger the observed specific cell signaling events through an interaction with PPM.

**Figure 3:**
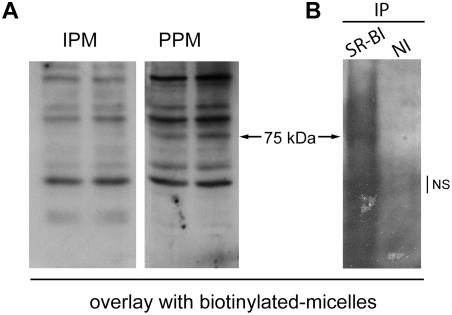
Ligand blotting assay showing specific interaction of PPM with Caco-2/TC7 homogenates and with SR-BI/CLA-1. (A) Caco-2/TC7 cell homogenates (150 µg) were subjected to SDS-PAGE and transferred onto nitrocellulose membranes. Membranes were incubated with PPM or IPM containing biotinylated phospholipids coupled to horseradish-streptavidin peroxidase and developed using the ECL system. (B) Caco-2/TC7 cell homogenates were immunoprecipitated with anti-SR-BI antibodies or non-immune serum and the resulting protein samples were subjected to SDS-PAGE and transferred onto nitrocellulose membrane. The membrane was incubated with PPM as in (A). NI, non immune serum; NS, non specific signal.

To identify proteins that specifically interact with PPM, we tested the interaction of cell extracts with labelled phospholipid-containing PPM or IPM in a ligand blotting assay. While both types of micelles interacted with several proteins, these interactions were enhanced with PPM; PPM also interacted with one 75 kDa protein that IPM did not interact with (**Figure 3A**). Of the lipid receptors known to be localized at the apical membrane of enterocytes with a similar molecular weight is the scavenger receptor SR-BI (CLA-1 in Human) [22], which is expressed in the brush border membrane of enterocytes of the duodenum and jejunum and in Caco-2 cells [23]–[25]. Other candidates NPC1L1 and FABPm are 150 kDa [26] and 40 kDa [27] respectively, and FAT/CD36 [28] is not expressed in Caco-2 cells ([25], [29] and Carrière et al. unpublished results). To test whether SR-BI/CLA-1 interacts with PPM, Caco-2/TC7 cell homogenates were immunoprecipitated with anti SR-BI/CLA-1 antibodies and then assayed by ligand-blotting. Results presented in **Figure 3B** confirm that there is an interaction between PPM and SR-BI/CLA-1 in the immunoprecipitate.

**Figure 4:**
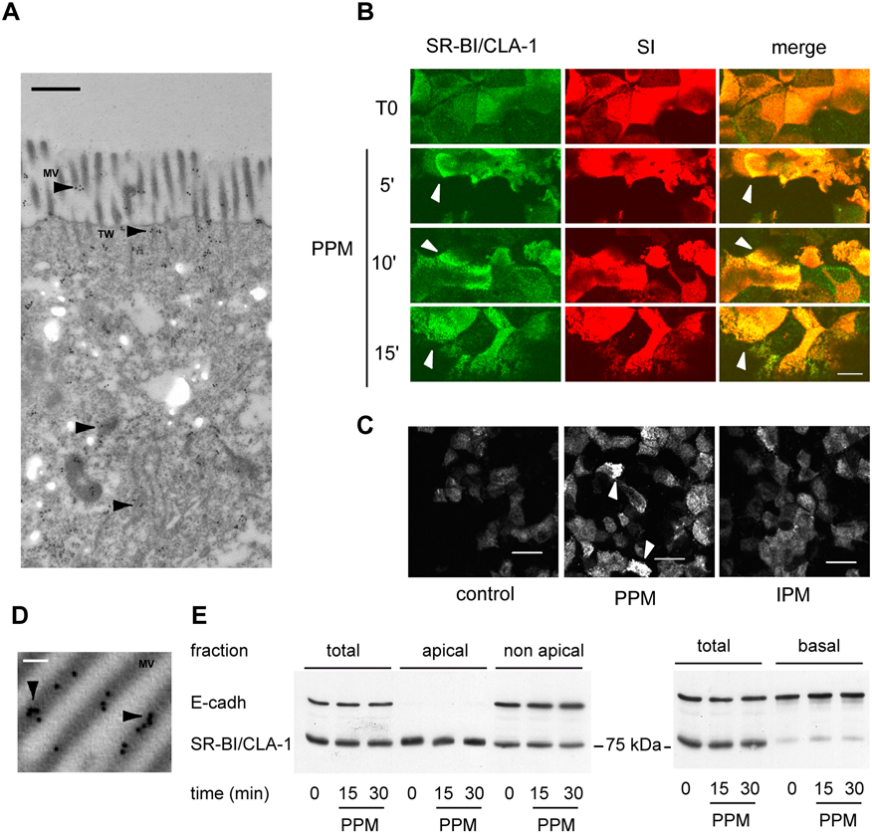
Subcellular localization of SR-BI/CLA-1 after the supply of postprandial micelles. (A) Immunoelectron micrograph of SR-BI/CLA-1 in untreated differentiated Caco-2/TC7 cells. MV, microvilli; TW, terminal web (bar, 0.5 µm). Note the significant amount of intracellular trafficking SR-BI/CLA-1 in addition to its main apical localization (arrowheads). (B) Immunolocalization of SR-BI/CLA-1 (green channel) and sucrase isomaltase (SI, red channel) in differentiated Caco-2/TC7 cells before (T0) and after 5, 10 and 15 min of apical PPM supply. Panels represent XY acquisitions at the apical level (bar, 10 µm). Arrowheads show clusters of SR-BI/CLA-1. (C) Immunolocalization of SR-BI/CLA-1 in differentiated Caco-2/TC7 cells in the absence (control) or presence of PPM or IPM for 20 min (bar, 20 µm). Arrowheads show clusters of SR-BI/CLA-1 (D) Immunoelectron micrograph of SR-BI/CLA-1 in Caco-2/TC7 cells supplied with PPM (MV, microvilli). Arrowheads indicate SR-BI/CLA-1 clusters (bar, 100 nm). (E) Cell surface biotinylation assay for apical SR-BI/CLA-1. Caco-2/TC7 cells were cultured in the absence (0) or presence of PPM for the indicated times. Cells were then selectively labeled with non-permeant biotin at the apical (left panel) or basal surface (right panel). Biotinylated fractions were obtained as described in Material and Methods. Total cell lysates (total), apical and basal biotinylated fractions (left and right panel respectively) and non-apical fractions (non-apical) were analyzed in immunoblots of SR-BI/CLA-1, E-cadherin being used as a basolateral membrane marker.

We then analyzed the effects of PPM on the expression of SR-BI/CLA-1. Immunoelectron microscopy showed that in control Caco-2/TC7 cells SR-BI/CLA-1 was predominantly localized at the apical pole of the cells, in microvilli and in the terminal web (**Figure 4A**), as described previously for pig and human enterocytes [24], [30]. Immunofluorescence analysis of SR-BI/CLA-1 localisation confirmed that SR-BI/CLA-1 co-localized with the brush-border marker sucrase-isomaltase in control Caco-2/TC7 cells, (**Figure 4B**). SR-BI/CLA-1 formed clusters at the apical membrane of enterocytes as soon as 5 min after PPM supply (**Figure 4B and 4D**). These clusters, which were still present after 20 min, were not observed in IPM-treated cells (**Figure 4C**). To determine whether SR-BI/CLA-1 clustering resulted from an increased recruitment at the apical membrane, cell surface biotinylation was assayed (**Figure 4E**). After incubation with or without PPM for 15 or 30 min, non-permeant biotin was added at the apex (**left panel**) or base (**right panel**) of the cells and biotinylated proteins were specifically isolated from the apical and basolateral cell membranes using a biotin-streptavidin complex. The amount of SR-BI/CLA-1 in total cell homogenates, in biotinylated fractions and in non-biotinylated fractions was compared by immunoblotting. There was much more SR-BI/CLA-1 at the apical membrane than at the basolateral membrane of enterocytes (**Figure 4E**, compare left and right panels). No increase in the apical amount of SR-BI/CLA-1 was observed after PPM supply (**Figure 4E, left panel**), indicating that PPM, while they did induce clusters of apical SR-BI/CLA-1, did not modify its targeting to the apical membrane of enterocytes.

**Figure 5:**
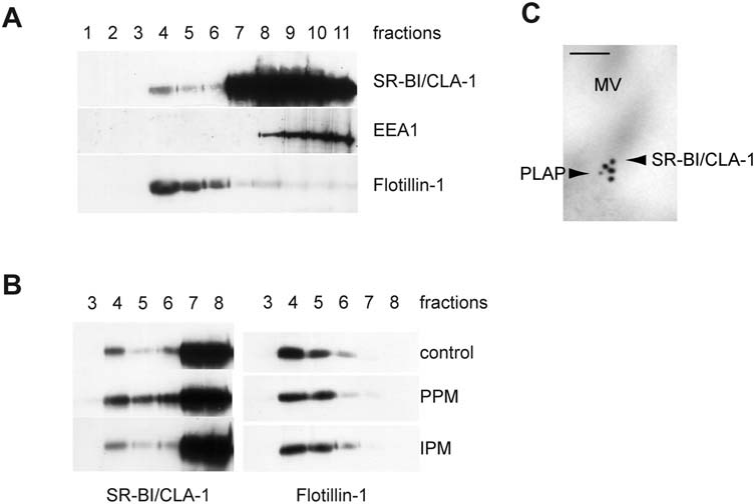
PPM supply induces movement of SR-BI/CLA-1 towards raft microdomains. (A) Caco-2/TC7 cells were harvested in the presence of Triton X-100 and the lysate fractionated on a 5–40% sucrose gradient. Eleven fractions were collected for immunoblots of SR-BI/CLA-1, EEA1 (early endosome antigen 1) and flottilin-1 (raft marker). (B) Caco-2/TC7 cells were cultured in the absence (control) or presence of PPM or IPM for 10 min and then harvested in the presence of Triton X-100. Cell lysates were applied to a 5–40% sucrose gradient and eleven fractions collected. Fractions 3 to 8 were analyzed by immunoblotting with antibodies against SR-BI/CLA-1 (left panel) and flottilin-1 (right panel). (C) Immunolocalization of SR-BI/CLA-1 and alkaline phosphatase (PLAP, used as raft marker) in the brush border of Caco-2/TC7 cells supplied with PPM. SR-BI/CLA-1 is labelled with anti-rabbit immunoglobulin-gold complexes (18-nm particles) and PLAP with anti-sheep immunoglobulin-gold complexes (12-nm particles). MV, microvilli; bar, 100 nm.

It has been reported that SR-BI function may depend on its localization in raft microdomains [30]–[32]. The effect of PPM or IPM supply on the association of SR-BI/CLA-1 with rafts was assessed by fractionating cell contents through a sucrose gradient (**Figure 5**). In cells that received no micelles in the apical medium, a large amount of trafficking SR-BI/CLA-1 was found in non-raft/high-sucrose density cell fractions (fractions 7–11), which contained the early endosome marker EEA1. However, SR-BI/CLA-1 was also localized in the low-sucrose density fractions (fractions 4–6) in raft microdomains identified by the expression of flotillin-1 (**Figure 5A**). When PPM were supplied to cells, more SR-BI/CLA-1 was observed in raft fractions (fractions 4–6) (**Figure 5B**). This localization was confirmed by immunoelectron microscopy (**Figure 5C**), which showed that after PPM supply SR-BI/CLA-1 was in the vicinity of the raft-associated alkaline phosphatase [33], [34]. No difference in the partitioning of SR-BI/CLA-1 was observed in cells supplied with IPM and control cells. PPM and IPM supply did not modify the subcellular distribution of flotillin-1 indicating that they did not alter properties of the sucrose gradient.

### SR-BI/CLA-1 is involved in PPM-induced cell signaling in enterocytes

**Figure 6:**
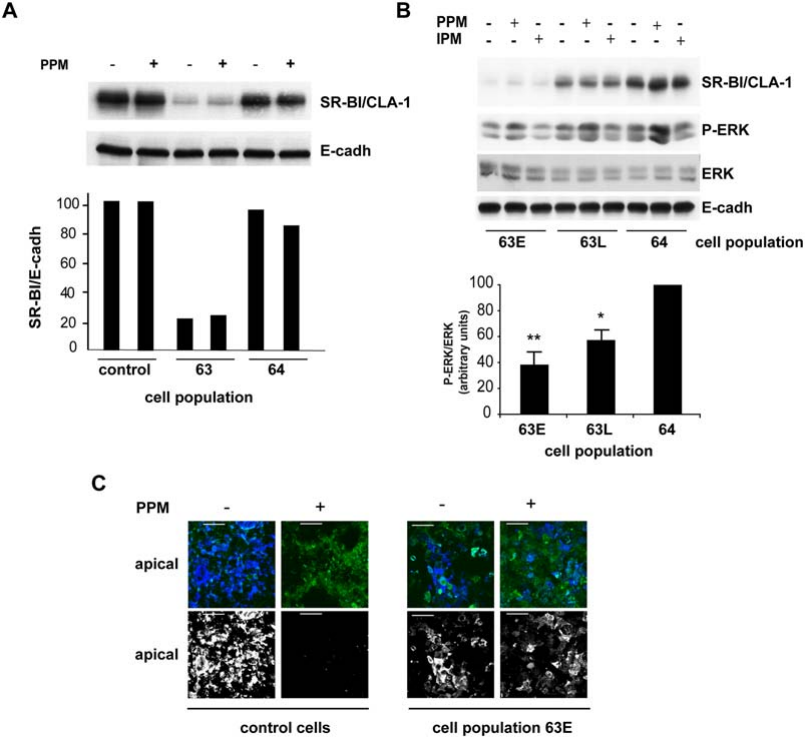
Knockdown of SR-BI/CLA-1 in Caco-2/TC7 cells impairs PPM-induced ERK1/2 phosphorylation and apoB chase. (A) Caco-2/TC7 Cell populations 63 and 64, expressing lentiviral shRNA 63 and 64 respectively, were analyzed at passage 4 after transfection in the absence of PPM or after 10 min of PPM supply. Cell lysates were analyzed by immunoblot with antibodies against SR-BI/CLA-1 and E-cadherin (E-cadh, used as loading control). The lower panel shows the level of SR-BI/CLA-1 expression normalized to the level of E-cadherin expression set at 100% for control Caco-2/TC7 cells. Results are from two independent sets of experiments. (B) Cell populations 63 and 64 were cultured on semi-permeable filters and incubated in the absence or presence of PPM or IPM in the apical compartment for the indicated times. An early (63E) and a late (63L) passage (corresponding respectively to passage 6 and 28 after transfection) of Cell population 63 were compared to Cell population 64 at passage 28. Cell lysates were analyzed in SR-BI/CLA-1 and phospho-ERK1/2 (P-ERK) immunoblots. Total ERK (ERK) and E-cadherin (E-cadh) were used as loading controls. Lower panel, the ratio of P-ERK expression normalized to total ERK expression in PPM-treated cells versus IPM-treated cells, set at 100% for Cell population 64. Results show the means±SEM of three independent sets of experiments. *P<0.05 compared to Cell population 64. (C) Control Caco-2/TC7 cells and an early passage of Cell population 63 (passage 7) were pre-incubated for 1 hour with cycloheximide before PPM were added for 20 min more. Immunolocalization of apoB in these cells was performed using concanavalin A (conA) to visualize the apical membrane. Upper panels show the merged labeling of conA (green) and apoB (blue) in the apical plane (XY planes; bar, 50 µm), lower panels show the same apical XY planes with apoB alone (white). Note the absence of apoB from the apical compartment of control cells after PPM supply resulting from its chase towards intracellular domains and the persistence of apoB at the apical pole of SR-BI/CLA-1-knockdown cells.

To determine how SR-BI/CLA-1 acts in PPM-induced cell signaling, we first analyzed the effect of SR-BI/CLA-1 knockdown in Caco-2/TC7 cells. Two different cell populations were obtained by stable lentivirus-mediated transfection of two different SR-BI/CLA-1 shRNA sequences. The expression of SR-BI/CLA-1 protein was 80% lower in Cell population 63 than in control Caco-2/TC7 cells, while Cell population 64 expressed SR-BI/CLA-1 protein at nearly the same level as control Caco-2/TC7 cells and was thus used as a negative control (**Figure 6A**). PPM supply did not modify the amount of SR-BI/CLA-1 protein in these transfected cell populations. After several passages, we observed that SR-BI/CLA-1 expression in Cell population 63 rose to 60% of that in control cells, whereas there was no change in the level of SR-BI/CLA-1 expression in Cell population 64 over the same period (**Figure 6B**). We used these differences in SR-BI/CLA-1 protein levels to analyze the relationship between PPM-dependent ERK1/2 phosphorylation and the amount of SR-BI/CLA-1 protein, i.e., by comparing Cell population 63 at early (20% expression) and late passages (60% expression) with control cells (100% expression, untransfected) and Cell population 64 (100% expression, transfected). As shown in **Figure 6B**, the PPM-induced ERK1/2 phosphorylation level was positively correlated with the amount of SR-BI/CLA-1, with the least ERK1/2 activation in the cell population expressing the least SR-BI/CLA-1. The PPM-dependent delocalization of the apical pool of apoB was compared in controls and Cell population 63 (at early passages). Cells were incubated in the presence of cycloheximide, which inhibits protein synthesis, so the disappearance of the preexisting apical pool of apoB (in the presence of PPM) resulting from its chase towards intracellular compartments could be visualized without confusion with the trafficking of newly synthesized apoB targeted to the apical membrane [14]. As shown in **Figure 6C**, in SR-BI/CLA-1-knockdown cells PPM supply did not stimulate the chase of apoB, which was maintained at the apical pole unlike control cells incubated with PPM, in which apoB disappeared from the brush-border domain.

**Figure 7:**
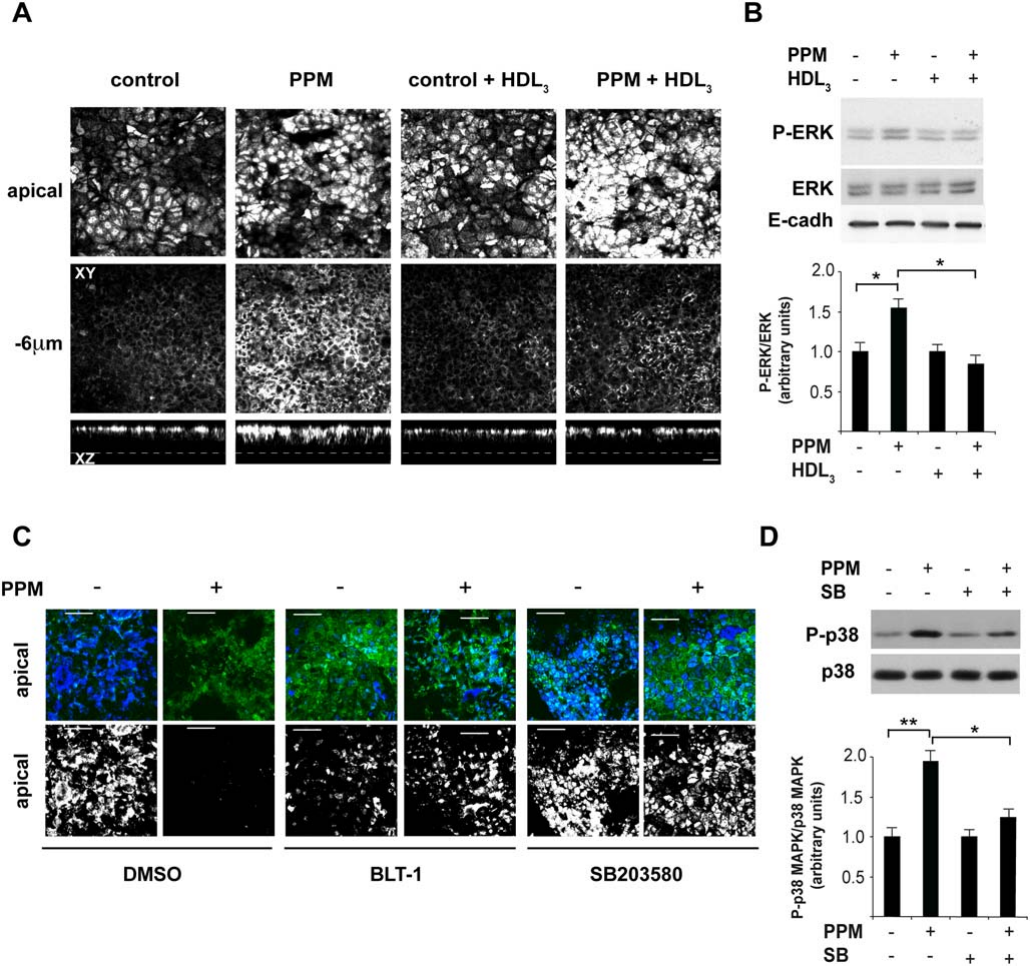
PPM-induced effects are impaired in the presence of competitors or inhibitors of SR-BI/CLA-1 and of the MAP kinase pathway. (A) Caco-2/TC7 cells were analyzed before and after a 10-min incubation with PPM with or without apical HDL_3_ (100 µg/ml). ApoB distribution was analyzed by confocal microscopy. Upper panels show XY acquisitions at the apical brush-border (BB) level and 6 µm below the apical plane. Lower panels show the corresponding XZ projections. Dotted lines indicate the base of the cells (bar, 20 µm). (B). Caco-2/TC7 cells were incubated with PPM and HDL_3_ as in (A). Cell lysates were analyzed in phospho-ERK1/2 (P-ERK) immunoblots with total ERK (ERK) and E-cadherin (E-cadh) levels used as loading controls. Lower panel, P-ERK levels normalized to total ERK expression. Results from two independent experiments are expressed as the mean±SEM, *P<0.05. (C) Caco-2/TC7 cells were pre-incubated for 1 hour with cycloheximide and 10 µM BLT-1 or 20 µM SB203580 or DMSO in both the apical and basal compartments. Inhibitors or DMSO were added again when PPM or DMEM were added in the apical compartment for 20 min. Immunolocalization of apoB in these cells using conA to visualize the apical membrane. Upper panels show the dual labeling of conA (green) and apoB (blue) in the apical plane (XY planes; bar, 50 µm), lower panels show the same apical XY planes with apoB alone (white). (D) Caco-2/TC7 cells were analyzed before or after a 10-min incubation with PPM with or without SB203580, a p38 MAPK inhibitor, added at the same time as PPM or DMEM. Cell lysates were analyzed in immunoblots of phospho-p38 MAPK (P-p38) and total p38 MAPK (p38). Lower panel, P-p38 MAPK levels normalized to total p38 MAPK levels. Results from two independent experiments are expressed as the means±SEM. *P<0.05, **P<0.01.

To further assess the involvement of SR-BI/CLA-1 in the cell signaling events that are specifically induced by PPM, we checked whether a ligand of SR-BI/CLA-1 could compete with PPM and interfere with their downstream effects, namely the phosphorylation of MAP Kinases and the apoB trafficking. HDL_3_, a strong SR-BI/CLA-1 ligand, was chosen as it has already been used as a competitor in other studies [35] including those using purified brush-border membrane vesicles of enterocytes [36]. The PPM-induced movement of the apical pool of apoB towards intracellular compartments (**Figure 7A**) and the phosphorylation of ERK1/2 (**Figure 7B**) were impaired in the presence of HDL_3_ at the apical pole of Caco-2/TC7 cells. By contrast, the addition of HDL_3_ in the absence of PPM had no effect compared to the controls. That SR-BI/CLA-1 has a role in PPM-dependent signaling is also reinforced by the fact that apoB trafficking is impaired in the presence of the inhibitor of SR-BI-dependent lipid transport BLT-1 [29], [37] (**Figure 7C**).

Finally, specific inhibition of p38 MAPK phosphorylation with SB203580 (**Figure 7D**) impaired the PPM-dependent apoB trafficking (**Figure 7C**), suggesting that the activation of p38 MAPK would be one of the mechanisms involved in the first step of TRL secretion.

## Discussion

Our previous studies led us to hypothesize that dietary lipids are sensed in their physiological form of postprandial micelles (PPM) by enterocytes [14]–[16], [38], a process that would trigger specific events leading enterocytes to secrete TG as TRL. Here we show that such a sensing system does exist in enterocytes and it involves the scavenger receptor class B type 1. We demonstrate that the enterocyte sensing of postprandial lipid micelles is characterized by the rapid delocalization of apoB, the structural protein necessary for the assembly of TRL, and by the rapid activation of ERK and p38 MAPK. In the absence of SR-BI/CLA-1, or in the presence of SR-BI/CLA-1 inhibitors or ligand competitors, these processes are impaired. This sensing step involves the clustering of SR-BI/CLA-1 at the apical membrane, a process associated with SR-BI/CLA-1 function in other cell models [39], and its recruitment into raft domains, that are known to contain various signaling molecules, including MAP kinases [40]. The localization of SR-BI/CLA-1 in raft domains differs from the observation of Hansen et al. (2003) [30]. This could be explained by differences in the experimental conditions used; these authors studied the lipid raft association of SR-BI in membrane preparations from intestinal mucosa scrapings of fasted pigs. One hypothesis is that the raft association of SR-BI depends on the nutritional status or on other environmental factors. It should be noted that the subcellular localization of SR-BI varies in different nutritional and hormonal conditions in adipocytes [41], [42].

SR-BI/CLA-1 has been extensively studied in terms of its involvement in cholesterol transport in the liver and in macrophages [43]. Recently, a role for SR-BI in lipid sensing in vascular endothelial cells has been described. In these cells, where SR-BI acts as a cholesterol sensor of HDL at the plasma membrane, the interaction with its ligand leads to a sequential activation of protein kinases that stimulate eNOS enzymatic activity [44], [45] and Rac GTPase activation [46]. The role of SR-BI/CLA-1 in the intestine remains controversial [47]–[50]. It was previously thought that SR-BI/CLA-1 was responsible for the absorption of micellar cholesterol from the intestinal lumen [24], [51], [52]. However, deficiency of intestinal cholesterol absorption is not observed in SR-BI knockout mice [53] and NPC1L1 was discovered to be the major intestinal transporter of cholesterol [54] together suggesting that SR-BI/CLA-1 may play another role in enterocytes. The newly assigned role of SR-BI/CLA-1 as a sensor of lipid micelles in enterocytes, described here, is in accordance with results showing that 1) SR-BI/CLA-1 binds not only high-density lipoproteins but also lipid structures devoid of apolipoproteins like PPM, 2) the three-dimensional structures of SR-BI/CLA-1 ligands are important in modulating its activity [51] and 3) various SR-BI/CLA-1 ligands trigger MAPK [35], [55] and PI3K/Akt [45] signaling pathways. Furthermore, the overexpression of SR-BI in the intestine leads to increases in cholesterol absorption and triglyceride secretion indicating a functional role for SR-BI in triglyceride lipid metabolism [50].

Our observations suggest that interaction between PPM and SR-BI/CLA-1 induces the establishment of a signaling platform in the brush border membrane of enterocytes that leads to the activation of cellular events, namely the activation of MAPK kinases and the trafficking of apoB, the structural protein involved in TRL assembly and secretion, from apical towards basolateral domains. A link has been made between apoB secretion and MAP kinase activation *in vivo* in fructose-fed hamsters [56]. In this insulin resistance model, characterized by overproduction of hepatic VLDL and intestinal apoB-containing lipoproteins, basal levels of phosphorylated ERK1/2 are higher in enterocytes and there is less intestinal apoB48 synthesis and secretion after inhibition of the ERK pathway [56]. More recently it has been shown that TNF-α-dependent overproduction of intestinal apoB-containing lipoproteins is mediated by the p38 MAPK pathway in hamster enterocytes [57]. The mechanism by which SR-BI/CLA-1-mediated PPM sensing leads to apoB delocalization and MAP kinase activation may also involve SR-BI/CLA-1 partners through protein-protein interactions. The C-terminal cytoplasmic domain of SR-BI/CLA-1 contains a PDZ-interacting domain. PDZK1 has been shown to be involved in the hepatic expression of SR-BI/CLA-1 [58] and PDZK1 knockdown in mouse models results in a decrease of SR-BI expression in the liver and intestine [59]. PDZK1 could thus mediate signaling events triggered by SR-BI/CLA-1.

That SR-BI may also participate to later intracellular events leading to TRL secretion, is suggested from a previous report by Hansen et al. (2003) [30]. In this study, pig intestinal explants were incubated with a lipid mixture containing corn oil, cholesterol, bile and pancreatin, i.e. mimicking the composition of lipid digestion products in the intestinal lumen, like the PPM used in our study. After three hours of incubation, SR-BI/CLA-1 was endocytosed from the brush border membrane of enterocytes and accumulated in newly formed cytosolic lipid droplets but not in secreted lipoproteins [30]. It should be recalled that, during the postprandial period, besides being secreted as TRL newly synthesized TG accumulate in cytosolic lipid droplets that can be remobilized and directed towards the endoplasmic reticulum lumen to be secreted as TRL, as observed in enterocytes *in vivo* [60], [61] and in Caco-2 cells [15], [62].

In conclusion, our results provide evidence for an enterocyte sensing of alimentary lipid-containing micelles, which could prepare these absorptive cells for the massive postprandial availability of lipids, and that SR-BI/CLA-1 participates early in this process at the apical membrane of enterocytes. Changes in PPM sensing, and in the resulting secretion of TRL, could be caused by factors such as diet, hormones or pharmaceutical compounds, which modify the expression and/or activity of SR-BI/CLA-1, a new target in studying how the early stages of the intestinal transfer of dietary lipids is controlled.
